# Healthcare access and mammography screening in Michigan: a multilevel cross-sectional study

**DOI:** 10.1186/1475-9276-11-16

**Published:** 2012-03-21

**Authors:** Tomi F Akinyemiju, Amr S Soliman, May Yassine, Mousumi Banerjee, Kendra Schwartz, Sofia Merajver

**Affiliations:** 1 Department of Epidemiology, University of Michigan School of Public Health, Ann Arbor, MI 48109, USA; 2 Cancer Control and Prevention Program, Michigan Public Health Institute, Okemos, MI, USA; 3 Department of Biostatistics, University of Michigan School of Public Health, Ann Arbor, MI 48109, USA; 4 Department of Family Medicine and Public Health Sciences and Barbara Ann Karmanos Institute, Wayne State University School of Medicine, Detroit, MI, USA; 5 Department of Internal Medicine, University of Michigan Medical School, Ann Arbor, MI 48109, USA; 6 University of Michigan Center for Global Health, Ann Arbor, MI 48109, USA; 7 Department of Epidemiology, University of Michigan School of Public Health, 1415 Washington Heights, Ann Arbor, MI 48109-2029, USA

**Keywords:** Mammography screening, Access to healthcare, Neighborhood effects, Socio-economic status

## Abstract

**Background:**

Breast cancer screening rates have increased over time in the United States. However actual screening rates appear to be lower among black women compared with white women.

**Purpose:**

To assess determinants of breast cancer screening among women in Michigan USA, focusing on individual and neighborhood socio-economic status and healthcare access.

**Methods:**

Data from 1163 women ages 50-74 years who participated in the 2008 Michigan Special Cancer Behavioral Risk Factor Survey were analyzed. County-level SES and healthcare access were obtained from the Area Resource File. Multilevel logistic regression models were fit using SAS Proc Glimmix to account for clustering of individual observations by county. Separate models were fit for each of the two outcomes of interest; mammography screening and clinical breast examination. For each outcome, two sequential models were fit; a model including individual level covariates and a model including county level covariates.

**Results:**

After adjusting for misclassification bias, overall cancer screening rates were lower than reported by survey respondents; black women had lower mammography screening rates but higher clinical breast examination rates than white women. However, after adjusting for other individual level variables, race was not a significant predictor of screening. Having health insurance or a usual healthcare provider were the most important predictors of cancer screening.

**Discussion:**

Access to healthcare is important to ensuring appropriate cancer screening among women in Michigan.

## Introduction

The breast cancer incidence-mortality disparity has been described as the disproportionately higher mortality rate among black women compared with whites in the United States but higher incidence among whites compared with blacks [1-6]. Between 2004 and 2008, breast cancer incidence rate was 133 per 100,00 among non-Hispanic whites compared with 120 per 100,000 among black women, while mortality rate was 23 per 100,000 among non-Hispanic whites compared with 32 per 100,000 among black women [7]. Similar trends have also been observed in Michigan [8]. Several studies have suggested that this disparity may be due to biological differences that predispose black women to more aggressive forms of breast cancer [4,9-13], while others attribute the disparity to differences in socio-economic status [5,14-17], access to healthcare [18,19] and mammography use [18,20]. Complete understanding of the incidence-mortality paradox observed in the US is complicated by the presence of racial disparities in these predictors.

In the United States, recent survival studies show that black women are more likely to be diagnosed with breast cancer at advanced stages compared with white women [15,18,19,21], and are less likely to have timely follow-up of abnormal mammography findings [22-24]. This suggests that the observed incidence-mortality paradox may be due to the lack of adequate mammography screening and diagnostic follow-up among black women [21]. Mammography screening remains the most effective method available to date and has been shown to increase the likelihood of early diagnosis, optimal treatment, and survival [25,26]. On the other hand, there are associated risks of overdiagnosis and overtreatment to healthy women which have also been discussed [27]. Nevertheless, the United States Preventive Task Force (USPTF) recommends biennial mammography screening for women ages 50-74 years [28]. However, data from hospital records in the US shows that mammography screening is underutilized, and racial disparities exist in screening [29]. For instance, the prevalence of mammography screening across the US in 2006 was 65% among whites, and 59% among blacks [1].

Previous studies have suggested that individual and neighborhood factors such as SES and healthcare access may contribute to underutilization and racial disparities in mammography screening [19,30-34]. For instance, adequate and timely mammography screening are more likely among women that are more educated, have higher income and have health insurance [35,36]. Research studies also suggests that residing in rural areas [30], and neighborhoods with low supply of primary care physicians and low number of health clinics was associated with lower likelihood of cancer screening [20]. In addition, residing in neighborhoods with low socio-economic status has also been associated with lower mammography screening rates [36,37]. Similarly, lack of adequate healthcare facilities at the neighborhood level may also have significant effects on the likelihood of getting adequate screening and follow-up [24,34]. Unfortunately, few studies in the US have comprehensively assessed the role of individual SES and access to care (such as health insurance and having a usual healthcare provider), as well as neighborhood level SES and access to care on the receipt of adequate breast cancer screening.

The aim of this analysis was to investigate individual (such as demographics, SES and health care access) and neighborhood (such as SES and health care access) determinants of mammography screening and clinical breast examination among adult women in Michigan, USA.

## Methods

### Data sources and analytic samples

The source of data for this analysis was the 2008 Michigan Special Cancer Behavioral Risk Factor Survey [38]. This was a disproportionate stratified telephone survey of non-institutionalized adults 40 years of age or older residing in Michigan, USA. Eligible individuals were identified using random digit dial sampling procedures to ensure that all residents had an equal chance of being included in the study. Within selected households that included at least one eligible adult, one adult was randomly selected to complete the interview. The aim of the survey was to assess risk factors, family history, screening behaviors and cancer knowledge among adults in Michigan. The survey questions were modeled after those of the Centers for Disease Control and Prevention Behavioral Risk Factor Surveillance System's telephone survey. The survey was designed to over-sample African Americans, Arab/Chaldean Americans, Asian Americans, Native Americans, and Hispanics residing in Michigan, USA in order to facilitate analysis of cancer screening and risk behaviors among these groups, and to ensure enough sample size for meaningful comparisons between these groups and the remaining Michigan population. To account for the varying probabilities of selection, sampling weights were applied to the dataset.

The analytic dataset was restricted to black and white women ages 50-74 who participated in the survey. There were 1163 women residing in 80 out of the 83 counties in Michigan that met this criterion. Michigan is one of 50 states in the US, with a population of about 10 million in 2008. The average county population size in 2008 was 120,512 [39]. Individual counties were identified using the US Federal Information Processing Standard (FIPS) code [40] to link county variables with individual cases' county of residence at diagnosis as reported in the survey. All analytic variables were based on self-reports from survey respondents.

Ethical approval was received from the Michigan Department of Community Health Institutional Review board, and informed consent was obtained from all participants.

### Data management

Race was categorized as black (n = 327) and non-Hispanic white (n = 836). Mammography use was characterized as the amount of time since the most recent mammography. For the analysis, mammography was categorized as received within the past two years (up to 24 months ago) or received more than two years ago. Clinical breast examination (CBE) was characterized as breast examination by a professional (doctor, nurse or other health professional) for lumps or other signs of breast cancer. CBE was categorized as received within the past two years or more than two years ago.

Adjusted Prevalence of Mammography Screening and CBE: estimates of mammography screening and CBE among black and white survey respondents were adjusted for misclassification bias using sensitivity and specificity values obtained from a published meta-analysis of previous studies [1,29]. The meta-analysis was based on 12 published studies that compared self-reported cancer screening such as mammography, CBE and Pap smear against medical and billing records among women ages 40 years and above. To assess for differential misclassification by race, sensitivity and specificity values were calculated separately for blacks and whites when data were available. Specificity was defined as the probability that a negative screening history was reported as negative; sensitivity was defined as the probability that a positive screening history was reported as positive. For mammography screening, sensitivity for both black and white women was 0.97, however, the specificity for black women was 0.49 and for white women was 0.62. For CBE, data was not available by race but overall, sensitivity was 0.94 and specificity was 0.25. In this study, adjustment for misclassification in mammography and CBE screening prevalence was done using the formula: *(estimated prevalence *- *1 + specificity) */ *(sensitivity + specificity 1 ) *[41].

#### Health care access

Individual health care access was assessed by including variables related to health insurance, having a usual source of care such as a particular hospital or clinic, and having a usual healthcare provider such as a personal doctor. We adjusted for differences in population size by dividing the counts per 10,000 people in each county. The derived variables were subject to principal component analysis (PCA) on count per 10,000 individuals of number of hospitals, number of medical doctors, number of medical doctors with obstetrics and gynecology specialty, number of DOs, number of DOs with obstetrics and gynecology specialty, number of nurse practitioners, and the number of mammography facilities. SAS Proc Factor was used to generate county-specific scores based on our input variables [42]. The first two components together accounted for 50.5% of the total variance. Based on this criterion, MDs per 10,000, MDs in Ob-Gyn per 10,000, nurse practitioners per 10,000, and DOs per 10,000 were loaded on the first component; hereafter named Personnel. Number of hospitals per 10,000 and number of mammography facilities per 10,000 were loaded on the second component; hereafter named Facilities. These scores were categorized into two groups; low and high.

#### Socio-economic status

County level socio-economic status was defined using PCA to create three composite measures related to affluence, disadvantage and immigration. Concentrated affluence was based on county level proportion of individuals making over $100,000, proportion over 25 years old with a college degree and the proportion of white collar workers. Concentrated disadvantage was based on county level proportion of persons in poverty, proportion of families with a female-headed household, proportion of households that are food stamp recipients, and proportion of unemployed. Concentrated immigration was based on the proportion of foreign born individuals and the proportion of non-English speaking individuals. The affluence, disadvantage and immigration scores accounted for 84%, 74% and 94% of the total variance respectively, in the data. These scores were categorized into two groups; low and high.

### Statistical analysis

All statistical analyses were performed using SAS statistical software (SAS, Version 9.2). Weights were applied to the descriptive analysis to facilitate the comparison of blacks and non-Hispanic whites, adjust for non-response and stratifications while taking advantage of the oversampling of black participants. Descriptive statistics were generated using chi-square statistics. Multilevel logistic regression models assessing significant individual and neighborhood predictors of recent mammography screening and recent CBE were created using Proc Glimmix in SAS. This procedure was chosen to account for the correlation of observations within counties. Two models were created; the first model assessed individual level predictors including age, race, income, employment, education, marital status, health insurance, having a usual source of care and having a usual healthcare provider. The second model included variables related to county level SES, health care access and proportion of blacks. The effect of these variables on the estimate of the intercept (county effect) was observed, and if found to be attenuated was taken as evidence of mediation.

## Results

Overall, 90.80% of black and non-Hispanic white women ages 50-74 had received a mammography test in the past 2 years; 90.98% of white women and 90.35% of blacks. After adjusting for misclassification, 85% of black women and 90% of white women had received a mammography test. 88.75% of women overall had received a CBE in the past 2 years; 88.53% of white women, 89.31% of black women. After adjusting for misclassification, 74% of black women and 68% of white women had received a CBE in the past 2 years.

Table 1 presents the distribution of survey respondents according to the SES and healthcare access characteristics of their county of residence. 49% of respondents resided in counties with low healthcare facilities compared with 51% in counties with the high healthcare facilities; 48% of respondents resided in counties with the low healthcare personnel compared with 52% in counties with low healthcare personnel. 43% of respondents resided in counties with a low proportion of affluent households, compared with 57% in counties with a high proportion. 61% of respondents resided in counties with a low proportion of socio-economically disadvantaged households compared with 39% in counties with the highest proportion. Furthermore, 70% of survey respondents resided in counties that were both high in concentrated affluent and concentrated disadvantage (data not shown). 53% of respondents resided in counties with less than 6% of immigrants, compared with 47% in counties with the over 6% of immigrants.

**Table 1:** Distribution of County Level Socio-Economic Status and Healthcare Access Variables (%) of 2008 SCBRFS Participants from 83 Michigan Counties, 2000

	Facilities^a^	Personnel^a^	Affluence^b^	Disadvantage^c^	Immigration^d^	Percent Black^e^
**Low**	49.35	48.07	42.57	61.04	53.05	35.52
**High**	50.65	51.93	57.43	38.96	46.97	64.48

^a^Personnel and Facilities, two measures of health care access were defined using principal components analysis on the count per 10,000 population of several variables by county: hospitals, mammography facilities, MDs, DOs and nurse practitioners

^b^Concentrated affluence was based on PCA of county level proportion of individuals making over $100,000, proportion over 25 years with a college degree and the proportion of white collar workers

^c^Concentrated disadvantage was based on PCA of county level proportion of persons in poverty, proportion of families with a female-headed household, proportion of households that are food stamp recipients, and proportion of unemployed

^d^Concentrated immigration was based on PCA of the proportion of foreign born individuals and the proportion of non-English speaking individuals

^e^Percent black was categorized as low if less than 6% and high if 6% or more

The distribution of individual demographics, SES and healthcare access variables, as well as county level SES and healthcare access variables by race are presented in Table 2. In general, blacks were more likely than whites to earn less than $35,000 per year, were less likely to be employed, and were more likely to be single, divorced or widowed (p < 0.001). In addition, blacks were more likely to have a usual source of care (such as a particular hospital or clinic), were more likely to be smokers, and more likely to have had a past diagnosis of breast cancer (p < 0.05). At the county level, 80% of blacks resided in counties with low healthcare facilities, although over 95% of blacks resided mostly in counties with high healthcare personnel. This is in contrast with white respondents who were more likely to reside in counties with high level of healthcare facilities (63%), but also in counties with the low healthcare personnel (65%). About 44% of white respondents resided in counties with the high concentrated affluence compared with 91% of blacks (p < 0.001). However, 83% of black respondents also resided in counties with high concentrated disadvantage compared with only 22% of whites. Blacks were more likely to reside in counties with high concentrated immigration (86%) compared with whites (32%). Over 99% of blacks resided in counties with over 6% of blacks compared with 51% of whites.

**Table 2:** Characteristics of 2008 SCBRFS Participants

Characteristic	White (N = 836)	Black (N = 327)	P-Value
**Age**			
50-60	52.74	55.99	0.32
61-74	47.26	44.01	

**Income ($)**			
< 35,000	31.86	40.98	< 0.001
35,000-74,999	32.37	23.83	
> 75,000	17.81	9.53	
Missing	17.98	25.66	

**Employment**			
Employed	47.38	36.13	< 0.001
Unemployed	18.07	15.31	
Retired/Unable	34.55	47.88	

**Education**			
Less than High School	41.05	40.86	0.48
Some College	34.82	36.28	
College plus	24.01	22.28	

**Marital Status**			
Single	2.46	13.40	< 0.001
Married	77.56	31.16	
Divorced/Separated	11.79	29.00	
Widowed	8.10	25.46	

**Usual Source of Care**			
Yes	90.28	95.47	0.001
No	9.69	3.99	

**Usual Healthcare Provider**			
Yes	91.35	91.42	0.24
No	8.59	8.04	

**Family History of Breast Cancer**			
Yes	18.68	16.38	0.35
No	80.96	83.62	

**Past Diagnosis of Breast Cancer**			
Yes	5.42	10.04	0.02
No	94.21	89.72	

**Health Insurance Status**			
Yes	92.85	92.33	0.09
No	7.15	7.12	

**Smoking**			
Yes	43.92	56.77	< 0.001
No	56.08	42.77	

**Facilities^a^**			
Low	37.48	79.66	< 0.001
High	62.52	20.34	

**Personnel^a^**			
Low	65.17	4.42	< 0.001
High	34.83	95.58	

**Concentrated Affluence^b^**			
Low	55.85	8.69	< 0.001
High	44.15	91.31	

**Concentrated Disadvantage^c^**			
Low	78.35	16.85	< 0.001
High	21.65	83.15	

**Concentrated Immigration^d^**			
Low	68.21	14.36	< 0.001
High	31.79	85.64	

**Percent Black**			
< 6%	49.15	0.73	< 0.001
> = 6%	50.85	99.27	

**Recent Mammography^e^**			
Yes	87.57	89.14	0.09
No	8.68	9.52	
Missing	3.76	1.34	

**Recent CBE^f^**			
Yes	86.82	86.24	0.29
No	11.25	10.32	
Missing	1.93	3.44	

**Follow-up Time^g^**			
< 2 weeks	26.69	25.12	0.01
> = 2 weeks	11.16	5.86	
No follow-up required	62.15	69.02	

^a^Personnel and Facilities, two measures of health care access were defined using principal components analysis on the count per 10,000 population of several variables by county: hospitals, mammography facilities, MDs, DOs and nurse practitioners

^b^Concentrated affluence was based on PCA of county level proportion of individuals making over $100,000, proportion over 25 years with a college degree and the proportion of white collar workers

^c^Concentrated disadvantage was based on PCA of county level proportion of persons in poverty, proportion of families with a female-headed household, proportion of households that are food stamp recipients, and proportion of unemployed

^d^Concentrated immigration was based on PCA of the proportion of foreign born individuals and the proportion of non-English speaking individuals

^e^Recent mammography defined as the receipt of a mammography test in the past 2 years

^f^Recent CBE defined as the receipt of a clinical breast examination in the past 2 years

^g^Follow-up time defined as the number of days before receipt of final results among women with an abnormal breast exam in the past 10 years

The results of the adjusted analyses predicting recent mammography screening and recent clinical breast examination are presented in Tables 3 and 4 respectively. As presented in Table 3, after adjusting for individual demographic variables such as age, income, employment, marital status and education, there was no statistically significant difference in mammography screening between black and white women (OR = 0.63, 95% CI = 0.34-1.16). In addition, having no usual source of care reduced the likelihood of mammography screening by 54% (OR = 0.46, 95% CI = 0.21-0.99); having no healthcare provider (such as a usual doctor or nurse) was associated with 68% lower likelihood of receiving a mammography test in the past two years (OR = 0.32, 95% CI = 0.15-0.69); and having no healthcare insurance was associated with a 73% reduction in the likelihood of receiving a mammography test (OR = 0.27, 95% CI = 0.14-0.54). Adjusting for county level SES and healthcare access covariates tended to attenuate individual level healthcare access variables, although all except having a usual source of care remained statistically significant; having no usual healthcare provider and no healthcare insurance remained the significant predictors of recent mammography use.

**Table 3:** Multivariate Multilevel Models for Recent Mammography by Individual and County Level Characteristics

Characteristic	Model 1: Demographics ^a^	Model 2: + County^ b^
**Race**		
White	0.63 (0.34-1.16)	0.64 (0.32-1.28)
Black (Ref.)	-	-

**Age**		
50-60	1.16 (0.71-1.91)	1.12 (0.68-1.85)
61-74 (Ref.)	-	-

**Income**		
< 35,000	0.33 (0.14-0.76)**	0.32 (0.14-0.74)**
35,000-74,999	0.47 (0.21-1.05)	0.46 (0.21-1.03)
> 75,000 (Ref.)	-	-

**Employment**		
Unemployed	0.85 (0.46-1.56)	0.90 (0.48-1.67)
Retired/Unable	1.15 (0.66-2.00)	1.13 (0.65-1.99)
Employed (Ref.)	-	-

**Education**		
Less than High School	2.19 (1.21-3.96)**	2.05 (1.12-3.75)*
Some College	1.49 (0.85-2.60)	1.45 (0.82-2.56)
College plus	-	-

**Marital Status**		
Single	0.50 (0.25-1.02)	0.51 (0.25-1.03)
Divorced/Separated	0.74 (0.43-1.27)	0.75 (0.43-1.31)
Widowed	1.24 (0.62-2.48)	1.22 (0.60-2.44)
Married (Ref.)	-	-

**Usual Source of Care**		
No	0.46 (0.21-0.99)*	0.46 (0.21-1.02)
Yes (Ref.)	-	-

**Usual Healthcare Provider**		
No	0.32 (0.15-0.69)**	0.33 (0.15-0.71)**
Yes (Ref.)	-	-

**Health Insurance Status**		
No	0.27 (0.14-0.54)**	0.26 (0.13-0.52)**
Yes (Ref.)	-	-

**Facilities^ c^**		
Low		1.55 (0.76-3.14)
High (Ref.)		-

**Personnel ^c^**		
Low		1.16 (0.48-2.80)
High (Ref.)		-

**Concentrated Affluence ^d^**		
Low		2.23 (0.89-5.56)
High (Ref.)		-

**Concentrated Disadvantage^ e^**		
Low		1.26 (0.55-2.87)
High (Ref.)		-

**Concentrated Immigration ^f^**		
Low		0.82 (0.32-2.14)
High (Ref.)		-

**Percent black ^g^**		
< 6%		0.88 (0.37-2.09)
> = 6% (Ref.)		-

^a^Model adjusting for individual demographic variables only

^b^Model adjusting for county level variables including healthcare access and SES in addition to individual demographic variables

^c^Personnel and Facilities, two measures of health care access were defined using principal components analysis on the count per 10,000 population of several variables by county: hospitals, mammography facilities, MDs, DOs and nurse practitioners

^d^Concentrated affluence was based on PCA of county level proportion of individuals making over $100,000, proportion over 25 years with a college degree and the proportion of white collar workers

^e^Concentrated disadvantage was based on PCA of county level proportion of persons in poverty, proportion of families with a female-headed household, proportion of households that are food stamp recipients, and proportion of unemployed

^f^Concentrated immigration was based on PCA of the proportion of foreign born individuals and the proportion of non-English speaking individuals

*P < 0.05, **p < 0.01, ***p < 0.001; Ref, reference group

**Table 4:** Multivariate Multilevel Models for Recent CBE by Individual and County Level Characteristics

Characteristic	Model 1: Demographics ^a^	Model 2: + County ^b^
**Race**		
White	0.67 (0.41-1.11)	0.68 (0.37-1.26)
Black (Ref.)	-	-

**Age**		
50-60	1.30 (0.84-2.01)	1.31 (0.85-2.03)
61-74 (Ref.)	-	-

**Income**		
< 35,000	0.49 (0.24-0.99)*	0.51 (0.25-1.03)
35,000-74,999	0.80 (0.40-1.57)	1.82 (0.41-1.62)
> 75,000 (Ref.)	-	-

**Employment**		
Unemployed	0.69 (0.40-1.18)	0.69 (0.40-1.20)
Retired/Unable	0.91 (0.55-1.49)	0.89 (0.54-1.47)
Employed (Ref.)	-	-

**Education**		
Less than High School	1.19 (0.69-2.04)	1.25 (0.72-2.16)
Some College	0.91 (0.54-1.53)	0.96 (0.56-1.62)
College plus	-	-

**Marital Status**		
Single	0.55 (0.29-1.04)	0.54 (0.28-1.04)
Divorced/Separated	0.84 (0.51-1.39)	0.82 (0.49-1.36)
Widowed	1.06 (0.59-1.91)	1.07 (0.60-1.93)
Married (Ref.)	-	-

**Usual Source of Care**		
No	0.55 (0.26-1.13)	0.53 (0.25-1.09)
Yes (Ref.)	-	-

**Usual Healthcare Provider**		
No	0.34 (0.17-0.67)**	0.35 (0.18-0.70)**
Yes (Ref.)	-	-

**Health Insurance Status**		
No	0.40 (0.21-0.75)**	0.37 (0.19-0.71)**
Yes (Ref.)	-	-

**Facilities^ c^**		
Low		1.04 (0.65-1.68)
High (Ref.)		-

**Personnel^c^**		
Low		0.65 (0.33-1.29)
High (Ref.)		-

**Concentrated Affluence^d^**		1.45 (0.70-3.00)
Low		-
High (Ref.)		

**Concentrated Disadvantage^e^**		1.61 (0.93-2.78)
Low		-
High (Ref.)		

**Concentrated Immigration^f^**		0.73 (0.34-1.58)
Low		-
High (Ref.)		

**Percent black^g^**		0.85 (0.45-1.62)
< 6%		-
> = 6% (Ref.)		

^a^Model adjusting for individual demographic variables only

^b^Model adjusting for county level variables including healthcare access and SES in addition to individual demographic variables

^c^Personnel and Facilities, two measures of health care access were defined using principal components analysis on the count per 10,000 population of several variables by county: hospitals, mammography facilities, MDs, DOs and nurse practitioners

^d^Concentrated affluence was based on PCA of county level proportion of individuals making over $100,000, proportion over 25 years with a college degree and the proportion of white collar workers

^e^Concentrated disadvantage was based on PCA of county level proportion of persons in poverty, proportion of families with a female-headed household, proportion of households that are food stamp recipients, and proportion of unemployed

^f^Concentrated immigration was based on PCA of the proportion of foreign born individuals and the proportion of non-English speaking individuals

*P < 0.05, **p < 0.01, ***p < 0.001; Ref, reference group

There were no significant racial differences in the model predicting receipt of recent clinical breast examination (OR = 0.68, 95% CI = 0.37-1.26) even after adjustment for demographics, county level SES and healthcare access covariates (Table 4). In addition, covariates that were significantly associated with a recent CBE included lacking a usual healthcare provider (OR = 0.34, 95% CI = 0.17-0.67) and lacking healthcare insurance (OR = 0.40, 95% CI = 0.21-0.75). After adjusting for county level SES and healthcare access, healthcare insurance was the only statistically significant predictor of recent CBE (OR = 0.37, 95% CI = 0.19-0.71).

## Discussion

Potential individual and neighborhood level determinants of adequate breast cancer screening among women residing in Michigan, USA were explored in this analysis. Race was not a statistically significant determinant of cancer screening in adjusted or unadjusted analysis; however, lack of healthcare insurance and lack of a usual healthcare provider reduced the likelihood of getting adequate mammography screening. These variables remained significant even after adjusting for county level SES and healthcare access. Similarly, lacking health insurance was associated with reduced likelihood of receiving a CBE in the past two years.

This study supports previous research that have found that individual healthcare access through health insurance and having access to a usual healthcare provider are important determinants of cancer screening [43-47]. We tested the hypothesis that county level SES and healthcare access would also significantly predict cancer screening even after adjusting for individual level predictors. None of our county level predictors were significant, and this may be due to several factors. Firstly, it is possible that by accounting for individual healthcare access, county level SES and healthcare access measures are no longer important. For instance, for someone who has health insurance and a usual healthcare provider, lack of healthcare facilities and high concentrated disadvantage in their county may no longer be relevant; they already have a relationship with healthcare personnel who may provide recommendations, send reminders and schedule tests. This is in line with another study which reported that having a physician recommendation was the single most important predictor of mammography use [48].

Secondly, other county-level covariates which were not assessed in this analysis may be stronger predictors of cancer screening such as community coherence. For instance, despite a lack of hospitals and/or mammography clinics, some communities with high internal coherence may work together to create community centers where cancer screening is offered periodically, or may encourage each other by traveling together to other neighborhoods to get screened. This positive impact of community coherence on health behaviors such as cancer screening has also been described extensively in the literature [46,49-52]. In addition, it is possible that measuring SES and healthcare access at the county level diluted our measures through the aggregation of smaller regions with high and low levels of the covariates. For instance, zip-code or census tract level measures may produce a more homogenous estimate of SES and healthcare access, leading to better estimates of neighborhood level estimates. Unfortunately, such data are not readily available and the only neighborhood level identifier in the SCBRFS dataset was the county of residence.

Some studies have suggested that older women with a personal history of breast cancer are less likely to receive surveillance mammography, especially women who had gone without a visit to a physician in the past year [53-55]. In addition, most of the women in this study did not require follow-up for an abnormal finding. However, among the women that did, the majority received the follow-up within two weeks. Research studies have also suggested that the most important predictors of diagnostic follow-up of an abnormal screening result were fear and anxiety, lack of access care and lack of information from screening staff [56,57]. Although we were unable to assess the impact of breast cancer history and follow-up time on mammography use or CBE due to the low sample size, these studies are in line with our findings that having a usual healthcare provider is a crucial predictor of breast cancer screening.

In examining racial disparities in mammography screening, it is important to include a discussion about the effectiveness of the program and the impact this might have on screening rates. A recent Cochrane library review advices that women should be informed that, for example, if 2000 women undergo regular screening for 10 years, 1 breast cancer death will be prevented, 10 healthy women will be wrongly diagnosed and treated, and 200 women will receive an initial wrong diagnosis that does not result in treatment [27,58]. Although we found no significant differences in health insurance status and having a usual healthcare provider between blacks and whites in this study population, this may not be true for the general population. This implies that the population group with better access to healthcare and a usual healthcare provider may benefit more from screening by having higher participation rates and a better understanding of the potential benefits. Black women, with a historical mistrust of the medical system, perceived racial discrimination or fear of a cancer diagnosis may be disproportionately harmed by the lack of accurate information on the benefits of screening [59]. This serves as further evidence that having access to healthcare and a usual healthcare provide may be the most important component to consider in eliminating racial disparities in breast cancer screening.

There are several strengths of this study that should be highlighted. First, the data was from a large, probability sample of older non-institutionalized adults residing in Michigan, improving the external validity of the results. Second, the survey was focused on understanding risk factors for cancer and modeled after national cancer risk factor surveys, thereby improving the quality of the questions and its internal validity. Third, the availability of a comprehensive set of individual socio-demographic and healthcare access variables enhanced the ability to control for potential confounders in the analysis. Fourth, the ability to incorporate a comprehensive measure of county healthcare access through several different databases including the FDA to assess mammography facilities improved the quality of the neighborhood covariates used in the analysis.

There are also a couple of limitations of this study. First, the level of misclassification observed in reports of cancer screening may apply to other questions in the survey, introducing potentially serious misclassification bias in the survey responses. However, such misclassification of socio-demographic and healthcare access variables, if present, is expected to be non-differential with respect to the outcome of cancer screening. In this study, adjusting for misclassification bias had a larger impact on reported clinical breast examination in the past two years compared with reported mammography use. For instance, adjusted CBE was 15 percentage points lower than reported among blacks, and 20 percentage points lower among whites. Adjusted mammography test prevalence was 5 percentage points lower among blacks, and less than 1 percent lower among whites. The observed misclassification may be due to lack of understanding of what CBE or mammography tests are. Other studies have suggested that when more descriptive or graphic descriptions of procedures are used, false positive rates decline [60]. In addition, misclassification bias has also been described as being related to 'forward telescoping of dates' in which events (such as screening) are remembered as occurring more recently than they actually did [29]. Secondly, we included county level healthcare access measures based on the quantity of healthcare facilities and personnel in a county. This county level aggregate analysis may not account for language, cultural or financial barriers to accessing such facilities.

In summary, our research suggests that individual healthcare access is an important determinant of adequate cancer screening among adult women in Michigan. Future studies in the US may focus on adjusting socio-demographic covariates for potential misclassification, as well as measuring neighborhood level effects at smaller geographical scales to further investigate the presence of racial disparities in cancer screening. Globally, more research studies should focus on identifying individual and neighborhood level factors that may influence cancer screening. As breast cancer incidence and mortality rates are projected to increase faster in the coming decades due to westernization of lifestyles and aging populations, early detection will become even more important. Understanding region-specific barriers to adequate screening will be helpful to design programs aimed at improving screening rates for women regardless of race/ethnicity, socio-economic status or neighborhood of residence.

## Competing interests

The authors declare that they have no competing interests.

## Authors' contributions

TA participated in the conception and design of the study, performed statistical analysis and drafted the manuscript. AS, MB, MY, SM and KS participated in the design of the study and helped to draft the manuscript. All authors read and approved the final manuscript.
